# Severe fever with thrombocytopenia syndrome virus: emerging novel phlebovirus and their control strategy

**DOI:** 10.1038/s12276-021-00610-1

**Published:** 2021-05-06

**Authors:** Mark Anthony Casel, Su Jin Park, Young Ki Choi

**Affiliations:** 1 College of Medicine and Medical Research Institute, Chungbuk National University, Cheongju, Republic of Korea; 2 Research Institute of Life Science, Gyeongsang National University, Jinju, Republic of Korea

**Keywords:** Viral infection, Infection

## Abstract

An emerging infectious disease first identified in central China in 2009, severe fever with thrombocytopenia syndrome (SFTS) was found to be caused by a novel phlebovirus. Since SFTSV was first identified, epidemics have occurred in several East Asian countries. With the escalating incidence of SFTS and the rapid, worldwide spread of SFTSV vector, it is clear this virus has pandemic potential and presents an impending global public health threat. In this review, we concisely summarize the latest findings regarding SFTSV, including vector and virus transmission, genotype diversity and epidemiology, probable pathogenic mechanism, and clinical presentation of human SFTS. Ticks most likely transmit SFTSV to animals including humans; however, human-to-human transmission has been reported. The majority of arbovirus transmission cycle includes vertebrate hosts, and potential reservoirs include a variety of both domestic and wild animals. Reports of the seroprevalence of SFTSV in both wild and domestic animals raises the probability that domestic animals act as amplifying hosts for the virus. Major clinical manifestation of human SFTS infection is high fever, thrombocytopenia, leukocytopenia, gastrointestinal symptoms, and a high case-fatality rate. Several animal models were developed to further understand the pathogenesis of the virus and aid in the discovery of therapeutics and preventive measures.

## Virology: An emerging tick-borne disease

Severe fever with thrombocytopenia syndrome (SFTS), a tick-borne infectious disease caused by a novel phlebovirus, is a growing global health concern. The main symptoms are high fever, low platelet and white blood cell levels, and gastrointestinal problems. The disease was first identified in China in 2009, and several SFTS epidemics have occurred in East Asia, with mortality rates of between 6 and 27 per cent. Researchers in South Korea led by Young Ki Choi at Chungbuk National University, Cheongju, review the latest research into SFTS virus and disease, including transmission, genetic diversity, epidemiology, pathology and clinical features, and pandemic potential. Animal models are helping researchers understand the virus and disease and develop treatments and vaccines.

## Introduction

The severe fever with thrombocytopenia-causing phlebovirus has been officially named *Dabie bandavirus*, which belongs to the genus bandavirus in the family *Phenuiviridae*, order *Bunyavirales*^1^. Synonymously this virus is known as Severe Fever with Thrombocytopenia Syndrome Virus (SFTSV) or *Huaiyangshan Banyangvirus*. *Dabie bandavirus* causes the clinical condition known as severe fever with thrombocytopenia syndrome (SFTS)^2^. Although the International Committee on Taxonomy of Viruses (ICTV) currently accepts the new nomenclature, the term SFTSV has been the most widely used. Therefore, in this article “SFTSV” and “SFTS” is used for the name of the virus and the disease, respectively.

SFTSV comprises a segmented, negative-strand RNA that includes large (L), medium (M), and small (S) segments^2^. The L segment encodes the RNA-dependent RNA polymerase (RdRp), which functions as the viral transcriptase/replicase. The M segment encodes a membrane protein precursor that matures into two glycoproteins, Gn and Gc, which constitute the envelope. The S segment is an ambisense RNA that encodes two proteins; the antisense RNA encodes Np and the sense RNA encodes NSs. Np functions in viral RNA encapsidation/formation of the RNP complex and NSs interfere with host interferon production^3,4^.

## Vector and disease transmission

The lifecycle and mechanisms underlying the sustained transmission of SFTSV in nature remain unclear, although transmission via ticks is considered the most probable route as other members of the *Phenuiviridae* family are vector-borne. The Asian longhorned tick, *Haemaphysalis longicornis (H. longicornis)*, acts as the main transmission vector of SFTSV^2^. The SFTSV RNA has also been detected in several tick species, including *Haemaphysalis flava, Rhipicephalus microplus, Amblyomma testudinarium*, *Dermacentor nuttalli, Hyalomma asiaticum*, and *Ixodes nipponensis*^5–8^ in SFTS endemic areas. Nonetheless, the virus itself has reportedly only been isolated from *H. longicornis* ticks^9,10^. As the detection of SFTSV in a tick species does not verify the capacity of the tick to act as a competent vector, further studies are needed to establish any of these other species as vectors. In addition, the transmission and infection capacity of SFTSV is determined by the ability of the tick species to adequately amplify and spread the virus to animals and humans. *H. longicornis* is endemic to the Asia-Pacific region and has a broad host range, including wild and domestic mammalian and avian species^11,12^. An additional characteristic of these tick species is that they can reproduce parthenogenetically and can survive in various environmental conditions^13^. Recently, *H. longicornis* have been detected in the United States of America where they appear to be rapidly spreading^11,14^. In addition, there have been reports of human bites by *H. longicornis* in the US^15^. As a result, tick surveillance studies have been conducted and, fortunately, SFTSV RNA has not yet been observed in *H. longicornis* ticks collected in the US^15,16^.

SFTSV is maintained in nature by either a tick-to-tick cycle (transovarial or transstadial virus transmission from adult to juvenile ticks or through co-feeding on the same host)^17^ or through a tick-to-mammal cycle (transmission during a blood meal on an infected animal)^18^. For the tick-tick cycle, ticks not only serve as a vector but also function as reservoirs of SFTSV. SFTSV has been detected in domestic and wild animals^19^; however, most vertebrate animals were found to be sub-clinically infected with SFTSV^17^. Hence, infected animals do not develop substantial viremia and long viremic periods, suggesting that these animals are not the natural vertebrate reservoirs, but instead might be an incidental host.

SFTSV can also be transmitted to humans through bites from SFTSV-carrying ticks, but humans are largely an accidental host. While the primary infection route to humans is through infected tick bites, cases of human-human transmission through direct contact with bodily fluids of SFTS patients have been reported^20,21^.

## Geographic distribution and genetic diversity

SFTS was first reported in China in 2009 and rapidly spread to other provinces in central, eastern, and northeastern regions^2^. Cases of SFTS were also reported in Japan^22^ and Korea^23^ in 2012 and recently in Vietnam^24^ and Taiwan^25^. Mechanisms for the expansion of SFTS remain unclear; however, the spread of emerging viruses is commonly attributed to two main mechanisms: increased contact between wildlife and human populations and geographical spread of hematophagous arthropod vector or their vertebrate host outside the area of endemicity. *H. longicornis* ticks are a common parasite of migratory birds known to breed and migrate between endemic areas in China, Korea, and Japan^2^. Moreover, the distribution of *H. longicornis* in the Asia-Pacific region matches the migration route of birds in the East Asian-Australasian flyway. This implicates migratory birds in the dissemination of *H. longicornis*^26^. However, further field studies are required to understand the association between tick vector habitat and the tick infestation rate of migratory birds in China, Korea, and Japan. Understanding the mechanisms of spread is vital for understanding the in-depth ecological epidemiology of this virus across its endemic range.

**Fig. 1:**
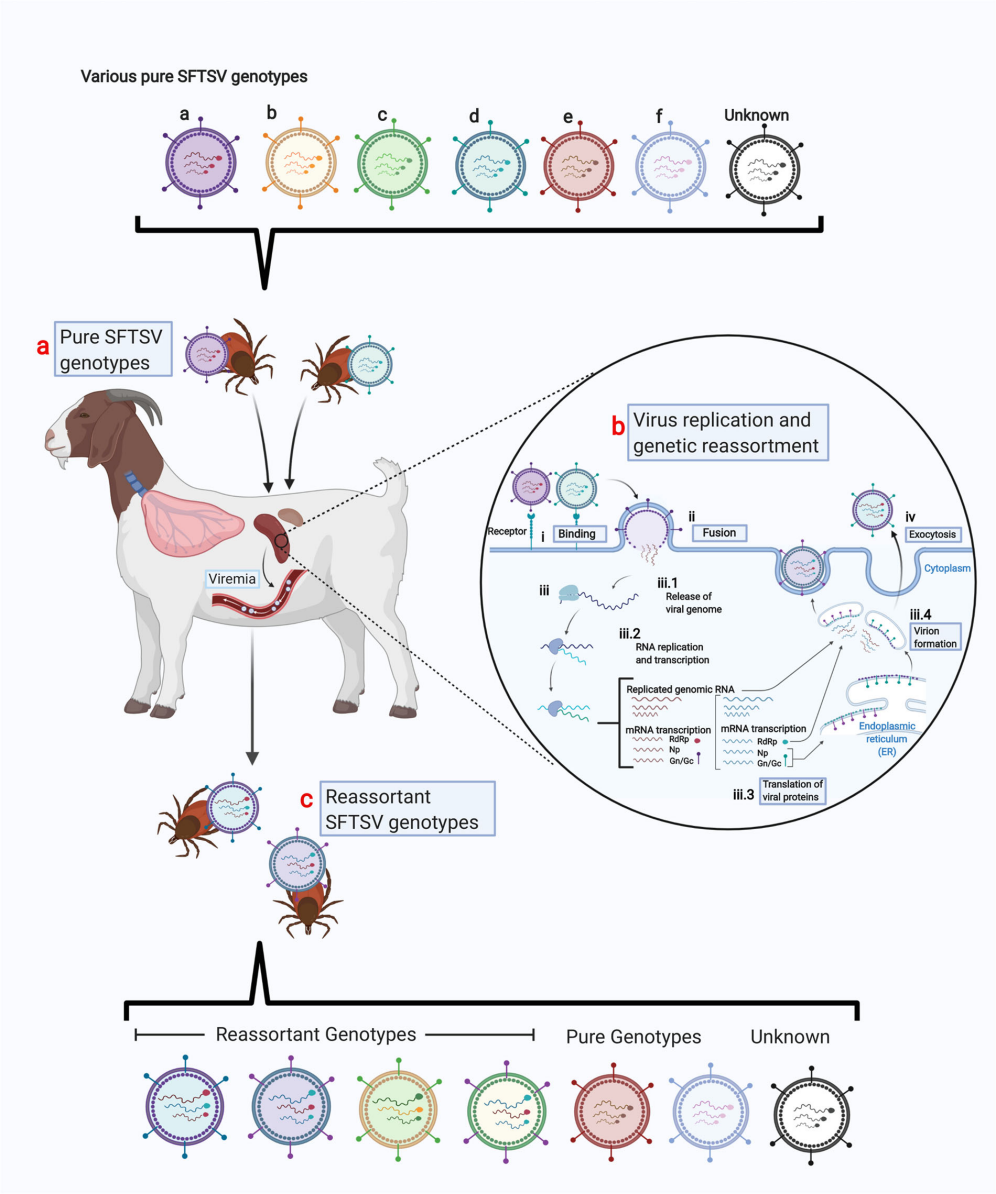
SFTSV genetic reassortment within a host. **a** Domestic and wild animals are readily infected with SFTSV from virus-carrying ticks during blood-feeding. Individual ticks may harbor different genotypes of SFTSV, thus causing superinfection to the vertebrate host. **b** Due to the segmented genome of the virus, the exchange of segments may happen between co-infecting SFTSV genotypes within the host cell during virus replication, generating a novel viral strain. **c** During the viremic period of the vertebrate host, ticks may acquire the virus during blood-feeding, further continuing the virus cycle and transferring the virus as it switches to a new vertebrate host (Created with BioRender.com).

Several papers have highlighted the genetic diversity of SFTSV^27–32^ caused by the segmented nature of the viral genome, which promotes frequent reassortment events. Thus, genetically diverse and novel genomic constellations have been generated in various hosts^33^. Further, SFTSV actively undergoes rapid evolution through gene mutations caused by the absence of a proofreading function during the viral RNA replication and transcription processes. To date, phylogenetic analysis of SFTSV strains has resulted in grouping into six genotypes, referred to as genotypes A to F, according to the suggestion of Fu et al.^31^, and the prevalence of SFTSV genotypes varies in different countries. Recently, Yun et al.^32^ demonstrated that B strains can be further subdivided into three different genotypes, B-1, B-2, and B-3. Further, there have been several reports of SFTSV reassortment in China, Korea, and Japan^27,32,34^, with at least nine different reassortant genotypes present in South Korea^32^ and seven SFTSV reassortants reported in China^31^. Thus, this indicates SFTSV strains actively undergo evolution in nature (Fig. 1).

The reported case fatality of SFTS varies greatly within affected countries throughout East Asia. Currently, Japan and South Korea reportedly exhibit high mortality rates of 27% and 23.3%, respectively. In contrast, SFTSV in China reportedly has a markedly lower mortality rate of 6.18%^35–37^. Interestingly, the most prevalent genotype circulating in Japan and Korea is the B-2 genotype, at a rate of 86% and 36.1%, respectively^32^. In China, the F genotype is most prevalent (43.6%) followed by the A genotype (20.1%)^31^. Thus, this suggests that the differences in the reported case-fatality rate might be associated with the differential distribution of SFTSV genotypes. Further supporting this idea, Yun et al.^32^ recently demonstrated the genotype-dependent pathogenesis potential of the virus. In this study, a close association between case fatality, patient age, and SFTSV genotype was noted. However, further study will be required to confirm that unique SFTSV genotypes cause differential pathogenesis.

## Seroprevalence of SFTSV in animals

Asian long-horned ticks go through three unique life stages (larva, nymph, and adult), and at each stage, they feed on a wide range of wild and domestic animals, including birds, livestock, and human companion animals^12^. The close interactions between humans and animals, especially in SFTSV-endemic areas, is speculated to increase the epidemic threat. This is due to the increased probability of tick bites as well as the potential for increased contact with secretions from SFTSV-infected animals^38,39^. In addition, the hypothesis that domestic animals may act as amplifying hosts of SFTSV^40,41^ there is a need to study the seroprevalence of this virus in animals.

SFTSV has been detected in a range of animal species (Table 1), including goats, sheep, cattle, dogs, pigs, chickens, cats, rodents, deer, boar, and hedgehogs^40^. A cross-sectional cohort study in China showed evidence of natural infection of SFTSV in domestic animals, with the highest seroprevalence in sheep (69.5%), cattle (60.4%), dogs (37.9%), and chickens (47.4%), and low prevalence in pigs (3.1%)^42^. A similar field investigation in SFTS endemic areas in China (Yiyuan, Shandong, and Jiangsu) revealed seroprevalence was highest among goats (57%) followed by cattle (32%)^43^. Differences in the rate of infection are likely dependent on the degree of exposure to virus-carrying ticks. Sheep, goat, and cattle are usually free-range, which renders them prone to tick infestations resulting in a higher probability of acquiring SFTSV infection. Intriguingly, while rodents are a known reservoir of Bunyaviruses the seroprevalence of SFTSV in rodents is lower than that of livestock^44^.

**Table 1:** SFTSV seroprevalence in animals.

Country	Animal species	Seroprevalence		References
		Antibody detection (%)	Carriage rate (%)	
China	Goats	57–83	9.1	^41,43,44,83,84^
	Sheep	69.5	–	^42^
	Cattle	28.18–73.7	26.3	^41,42,44,83,84^
	Dogs	6–37.9	–	^42,44,84^
	Pigs	3.1–6	–	^42,44,83,84^
	Chickens	1–47.4	–	^42,44,83,84^
	Goose	1.67	–	^44^
	Hedgehog	2.67–50	–	^41,44^
	Rodents	4.36	–	^44^
	Minks	8.4	–	^85^
South Korea	Goats	6.9–14.4	2–2.44	^86,87^
	Dogs	13.9	0.5	^28,88^
	Pigs	–	1.7	^86^
	Cats	–	0.2–4.76	^28,89,90^
	Wild boars	1.9	3.7	^90^
	Korean Water Deer	23.8	–	^90^
Japan	Outdoor Dogs	9.1	–	^91^
	Impounded Dogs	14.3	–	^91^
	Cattle	2.2	–	^92^
	Wild Boar	25–51	–	^91,93,94^
	Wild Deer	25	–	^91^

SFTSV antibodies have been detected predominantly in animal species with close associations to human life. These susceptible vertebrate hosts are needed for the establishment and maintenance of the arboviral transmission cycle. Whether prolonged or persistent infection can happen in SFTSV-infected animals is still unknown. Moreover, a limited number of SFTSV surveillance studies have been conducted in animals due to the rarity of clinical symptoms of SFTS^19^.

## Clinical characteristics in SFTS patients

### Clinical manifestation

Clinical symptoms of SFTSV infection, such as high fever and thrombocytopenia, were generally reported with a 7–14 (average of 9) day incubation period^45^. Besides high fever and thrombocytopenia, the main clinical manifestations include gastrointestinal disorders, leukocytopenia, and hemorrhagic tendency^46^. In addition, cases with atypical symptoms and asymptomatic SFTS infections were also reported^22^. Generally, the SFTS clinical course is characterized by three distinct periods based on disease progression: fever stage, multiple-organ dysfunction (MOD) stage, and convalescent stage^17^. During the fever stage, patients initially presented with acute high fever and high serum viral load with thrombocytopenia, leukopenia, and lymphadenopathy arising later in this phase. From the fever stage, this disease progresses to MOD, which in most cases develops approximately 5 days after disease onset. The MOD stage is characterized by hemorrhagic manifestations, neurologic symptoms, continued decline in platelet numbers, disseminated intravascular coagulation (DIC), and multi-organ failure leading to death^46^. Neurological symptoms include lethargy, muscular tremors, convulsions, and coma, which frequently occur in the terminal stage^47^. Patients with milder or self-limiting infection progress directly to the convalescence stage. Additional clinical aspects of SFTS include substantial elevation of serum levels of alanine aminotransferase (ALT), aspartate transaminase (AST), blood urea nitrogen (BUN), lactate dehydrogenase (LDH), creatinine kinase myocardial band fraction, and increased activated partial thromboplastin time (aPTT)^48^. These parameters are used to monitor heart, liver, and kidney function, and abnormal evaluations indicate organ injury and dysfunction.

### Senescence and severe clinical manifestations

Generally, elderly people with weakened immune function and chronic diseases tend to be most vulnerable to a severe viral illness. Furthermore, as age advances, the immune system changes in two major ways with the occurrence of immunosenescence and increase systemic inflammation. In immunosenescence, there is a gradual decline in immune function due to defects in both the innate and adaptive immune systems^49^. In addition, during aging, there is also a chronic increase in systemic inflammation, which arises from a hyperactive, yet ineffective, alert system^50^.

The majority of fatal SFTS cases occur in patients >50 years of age, making advanced age a risk factor associated with disease severity and fatality^51^. Further, the case fatality rate increases with the advancement of age in SFTS-afflicted patients^52^. While all age groups are susceptible to SFTSV, only geriatric patients have been found to succumb to infection^51^. This suggests that in SFTS aging is a determinant of morbidity and mortality.

### Poor prognostic factor

Numerous risk factors are associated with fatal outcomes of SFTSV infections^2,7,53^. Several clinical presentations and abnormalities in laboratory parameters (such as respiratory failure, hemorrhagic manifestations, DIC, and liver and kidney dysfunction) have also been considered to contribute to poor prognosis. Apart from the advanced age of the affected patient, hemophagocytic lymphohistiocytosis (HLH) and central nervous symptoms have also been found to be associated with fatal outcomes. Further, an association between fatal cases of SFTS and HLH was described in another study^54,55^, although only a small number of cases were reported. HLH is an immune-mediated life-threatening syndrome associated with excessive immune activation^56^. Encephalitis likely occurs due to the existence of SFTSV in the cerebrospinal fluid since there is no evidence of an alternative etiology^57^. Common manifestation includes headache, confusion, and seizure^45^. Owing to their neurotropic properties, other Bunyaviruses commonly cause symptoms within the central nervous system^58^. The development of these central nervous symptoms is frequently seen and consistently found to be associated with fatal outcomes^59^. Further, neurotropism of SFTSV was reported by Park et al.^60^ in a study where viral transcripts of SFTSV were found in the brain and spinal cord of an aged ferret model. However, the mechanisms underlying neurotropism of SFTSV remains to be defined.

Clinical manifestations in hospitalized SFTS patients vary from case to case. Hence, several scoring approaches have been proposed to assess the severity of infection and predict fatal outcomes for SFTS^48,61,62^. As the criterion differs depending on the insights of the authors, the accuracy and sensitivity of the proposed models will require further validation with increased patient numbers.

## Probable mechanism for SFTSV pathogenesis

Pathological studies are important for the understanding of viral pathogenesis. In addition, autopsies have provided valuable insight and knowledge of the pathogenic mechanisms of SFTS and how they relate to disease severity. For example, necrotizing lymphadenitis, especially in regional lymph nodes nearest to the insect bite wound, has been found to be pathognomonic for SFTS^63^. Besides necrotizing lymphadenitis, the involvement of a non-lymphoid organ is an important pathological feature of fatal SFTS; however, the source of infectious virus in the affected organs has not been defined^64^. Data presented by Suzuki et al.^65^ showed that while SFTSV was not present in the parenchymal cells, it was found in the capillaries of these non-lymphoid organs where infected B cells had infiltrated. B cells are the center of the adaptive humoral immune response and are responsible for mediating the production of antigen-specific immunoglobulins^66^. Immunoglobulin gamma (IgG) is essential for virus neutralization and viral clearance; however, in the case of fatal SFTS, patients fail to mount an IgG antibody response because of a failure in B-cell class switching^65^. This deficiency in adaptive immunity is due to the disruption of B-cell-mediated humoral immunity^67^. Further, analysis of fatal SFTS cases revealed that a large fraction of PBMC plasmablasts did not express IgM and IgG, explaining the inadequacy of the humoral B-cell response^67^. In addition, neutralizing IgGs were not detected in fatal SFTS cases, probably due to the inability of infected IgG-positive B cells to undergo class switching^67^. Collectively, these findings suggest that during the end stage of lethal SFTSV infection, differentiating B cells in the secondary lymphoid organs are the primary targets of the virus. Thus, B-cell susceptibility to SFTSV and the mechanisms of virus dissemination during the early stages of infection remain important topics to explore.

**Fig. 2:**
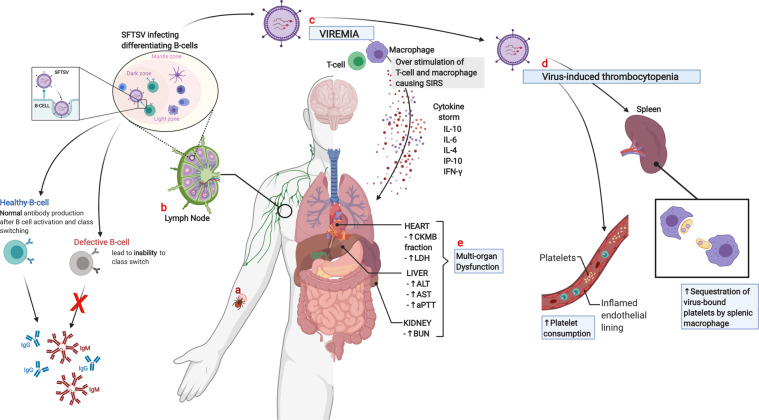
Probable mechanism of SFTSV pathogenesis. **a** SFTSV transmission to humans commonly occurs from virus-carrying-tick-bite. **b** The SFTSV then invades the lymph node nearest to the tick-bite wound, targeting immune cells such as B-cells, impairing host immune response from invading pathogen. **c** After further replication, the virus goes to the systemic circulation, in response to viremia, other immune cells are over-stimulated causing cytokine storm and severe inflammatory response syndrome (SIRS)^69^. **d** Thrombocytopenia is a hallmark of SFTSV infection; hence, several mechanisms may be attributed to the decrease of platelet count, such as, increase consumption of peripheral platelets from virus-induced activation of the coagulation pathway, which may be related to the occurrence of disseminated intravascular coagulation or due to endothelial damage from severe systemic inflammation^83^, and by clearance of SFTSV-bound-platelet by splenic macrophages^75^. **e** Disseminated intravascular coagulation and endothelial damage caused by cytokine storm lead to multi-organ dysfunction, reflected by the elevation of liver, kidney, and heart serum markers (Created with BioRender.com).

Cytokine storm has also been noted as a major pathological feature of patients with fatal SFTS^68^. Reports of cytokine and chemokine expression kinetics in patients with SFTS suggest interleukin (IL)-6, IL-10, interferon (IFN)- γ-induced protein (IP)-10, and IFN-gamma levels are generally elevated during the early phase of the disease^68–71^. Kwon et al.^70^ reported that IP-10 levels significantly correlate with viral load; however, due to the small sample size, further study is needed to confirm this. In addition, associations between cytokine/chemokine levels and platelet counts and serum chemistry (AST, LDH, ALT) have also been reported^72^. Decreased platelet levels were associated with soluble CD40 ligand and platelet-derived growth factor BB levels decreases. Similarly, a decrease in platelet levels was reported when IL-10, soluble IL-2 receptor alpha, and IP-10 levels increase. Ding et al.^68,72^ observed a positive correlation between serum AST, ALT, and LDH levels and several soluble molecules including IL-10, sIL-2RA, heat shock protein 70, IP-10, IL-4, IFN-γ, and tPAI-1. In addition, IL-10 hypersecretion was reported in fatal and severe SFTS, where it induced compensatory anti-inflammatory response syndrome, triggering abnormal and uncontrolled inflammatory dysregulation^73^ (Fig. 2).

## Pathology and pathogenesis in animal models

To understand the detailed disease profile associated with SFTS, many researchers have attempted to establish animal models that recapitulate the clinical features of SFTSV human infections. The development of an animal model is crucial not only for understanding the pathogenesis of SFTSV infection and how the immune system responds to such an assault, but also for the development of vaccines and therapeutics.

### Mouse model

Several young adult (5 to 8-week-old) immune-competent mouse strains (A/J, C57BL/6J, CAST/EiJ, DBA/1J, DBA/2J, FVB/NJ, NZBWF1/J, SIL/J, BXD68/RwwJ, BXD34/TyJ, and ICR CD-1) have been examined as potential models; however, none demonstrated lethality after SFTSV infection^74–76^. Likewise, aged mice (12–24 months) also failed to show lethality, even though increased age is a risk factor for severe SFTS^74^. However, several immunocompromised mouse models did exhibit lethality in response to SFTSV infection, including newborn mice, STAT-2^−/−^ mice, and interferon alpha/beta receptor knockout mice (IFNAR^−/−^) 129^77–79^. In addition, IFNAR^−/−^ and C57/BL6 mice recapitulate the hematologic manifestation that is similar to human cases such as lymphocytopenia and thrombocytopenia, however, the lethality of SFTSV were not observed in C57/BL6 mice^74,75^.

Currently, mouse models used to represent lethal SFTSV infection are IFNAR^−/−^, newborn, and mitomycin-treated immune-suppressed mouse. Studies in these animals have shed light on viral disease progression, the action of potential therapeutics, and vaccination approaches^80^, as these models exhibit pathological changes in SFTSV-targeted lymphoid and non-lymphoid organs. The fact that SFTSV causes severe disease in IFNAR^−/^^−^ mice suggest that type I interferon may be essential for host resistance to SFTS; however, the lack of immune components may substantially affect innate and adaptive immune responses after virus infection, complicating interpretation of the study results.

### Ferret model

While young ferrets do not show signs of disease, aged ferrets have previously been demonstrated to be susceptible to SFTSV-induced disease^60^. Aged ferrets demonstrate clinical symptoms characteristic of SFTS including thrombocytopenia and leukocytopenia, and viral RNA or antigens can be detected in serum, spleen, liver, and lung tissues^60^. Compared with young adult ferrets, transcriptional profile analyses of PBMCs from aged ferrets revealed that many severe and persistent inflammatory responses are mediated by activated immune-modulating cells. Thus, factors associated with aging (immunological and hematological dysfunction) may be required to recapitulate fatal outcomes in the ferret model. Despite the relevance of ferrets for use as a model of lethal SFTS, several factors limit their use, including low availability of aged animals and limited reagents for immunological studies.

### Non-human primate model

Rhesus macaques demonstrate mild features of SFTS infection and immune responses similar to less severe human SFTS cases^81^. Clinical symptoms observed using this animal model include slight febrile response; however, SFTSV infection in rhesus macaques did not cause fatality or severe clinical features (gastrointestinal symptoms, hemorrhages, and central nervous system symptoms) seen in severe human cases. Further, SFTSV infection of Cynomolgus macaques resulted in no visible clinical signs of infection and viral RNA could not be detected during the 14-day study period^74^. There is evidence that the non-fatal features observed in rhesus macaques are associated with the age of the animal. Rhesus macaques have a median lifespan of 27 years^82^ with juveniles being <5 years, adults 5 to <20 years, geriatric animals 20–25 years, and aged rhesus macaques being more than 25 years of age^82^. As the severity of SFTS is positively correlated with advanced age, further studies using aged macaques are needed to demonstrate the fitness of this animal model.

Choosing an animal model requires careful consideration, especially concerning the suitability of the animal for antiviral and vaccine studies. Thus, a detailed understanding of SFTSV pathogenesis in each model is pivotal, and differences and similarities between animal models and human cases must be considered carefully.

## General conclusions

SFTSV presents an impending threat to public health with pandemic potential and the ability to cause nosocomial transmission. As humans continue to encroach on wildland for agriculture and outdoor activities they become exposed to vector-carrying pathogens, which normally would remain in these areas. Thus, virus spillover events often happen at the interface between humans, animals, and the environment where ticks are endemic. Recent reports of the wider range of SFTS in Asia, together with the trans-regional dispersion of the competent vector further indicate the increasing epidemic and pandemic potential of SFTSV. Therefore, continuous surveillance for this pathogen is essential to address these concerns.

Several studies have investigated the complex interaction between SFTSV and the host immune system. For example, the failure to mount a virus-specific humoral response has been attributed to the dysfunctionality of B-cells. Further, it was also reported that differentiating B cells in secondary lymphoid organs are the primary target of the virus at the end stage of lethal SFTSV infection. Moreover, overexuberant immune response to this virus can contribute to progressive organ damage leading to death. Despite the current findings, additional studies are needed to further explain pathogenesis during the early phase of SFTSV infection and to define the mechanisms of pathogenesis that differ between fatal and surviving SFTSV infection cases. Extensive studies and development of animal models have been undertaken to gain further insight into SFTSV pathogenesis. The generation of a lethal animal model is pivotal for the development of preventive and therapeutic measures to combat this virus. Currently, no existing standard therapeutic protocol in combating SFTSV infection was established and commercial vaccines are none yet available. De novo development of new antiviral treatments is a promising approach but it is time and resources consuming. Thus, the repurposing of drugs has been increasingly conducted to find candidate treatment against emerging viruses, and several studies on repurposed drugs showed promising results. Vaccines are a powerful tool in preventing outbreaks from becoming a global crisis and the development of SFTSV vaccine is pivotal given that the disease is relatively novel. Several vaccine studies have been published, demonstrating protection efficiency using an animal infection model. While published vaccine studies reported to generate a robust antibody response, several challenges need to be addressed to successfully develop an effective vaccine, such as, a universal vaccine that can induce cross-protective immunity against different SFTSV genotypes, the establishment of a clinical trial design, and a standardized method to evaluate vaccine effectiveness including the vaccine’s safety profile. As the study of SFTSV is still in its infancy, many questions remain to be addressed. Future studies making use of technological advances will provide insight into how various components and systems work together to execute the complex biologic processes at work in this disease.
